# Chronic Sclerosing Osteomyelitis of the Mandible: A Case Report

**DOI:** 10.7759/cureus.76728

**Published:** 2025-01-01

**Authors:** Abdulsalam S Aljabab, Elaf M Algharbi, Sahar F Alotaibi, Abdullah M Almotreb

**Affiliations:** 1 Oral and Maxillofacial Surgery Department, King Fahad Medical City, Riyadh, SAU; 2 Oral and Maxillofacial Surgery Department, King Saud Bin Abdulaziz University for Health Sciences, Riyadh, SAU; 3 Oral and Maxillofacial Surgery Department, Riyadh Specialized Dental Center, Riyadh Second Health Cluster, Riyadh, SAU

**Keywords:** bone shaving, chronic recurrent multifocal osteomyelitis, chronic sclerosing osteomyelitis, fibro-osseous lesions, mandible, trismus

## Abstract

Chronic sclerosing osteomyelitis (CSO) is a rare, non-suppurative inflammatory condition of the bone characterized by progressive sclerosis and fibrosis, often requiring a multifaceted diagnostic approach. This case highlights an eight-year-old female with progressive mandibular swelling and trismus unresponsive to prior treatments. Imaging and histopathological evaluation revealed features consistent with CSO, excluding infectious or neoplastic causes. Intraoperative findings prompted a shift from planned segmental resection to conservative bone shaving, preserving mandibular integrity while improving jaw mobility. Postoperative outcomes were favorable, with no evidence of new lesions or complications. This case underscores the importance of individualized management strategies and further research to optimize diagnostic and therapeutic protocols for pediatric CSO.

## Introduction

Chronic sclerosing osteomyelitis (CSO) is an uncommon, long-term inflammatory condition of the bone that leads to persistent thickening (sclerosis). Unlike other forms of osteomyelitis, it does not produce pus, involve sequestered bone fragments, or cause fistulas. The condition is thought to stem from nonbacterial or autoinflammatory processes, though its precise cause remains uncertain [1-6]. Originally described by Carl Garré in 1893, CSO frequently affects the mandible but can also occur in other bones, particularly long bones. Clinical signs often include chronic, progressive pain, localized swelling, and in some cases, joint stiffness or difficulty opening the mouth (trismus), depending on the site involved [2,7].

Radiological evaluations show a progression from periosteal reactions and small bone lesions in the early stages to significant bone sclerosis in later stages. Histological findings often reveal chronic inflammation, fibrosis, and evidence of bone remodeling [1-5]. Diagnosing CSO involves excluding infectious causes through advanced imaging techniques, such as bone scintigraphy and anti-granulocyte antibody imaging, which helps confirm the absence of bacterial involvement.

Management primarily relies on anti-inflammatory therapies, including corticosteroids and bisphosphonates. Surgical interventions, such as decortication or intramedullary nailing, may be necessary for severe or recurrent cases but carry a risk of relapse. Physical therapy is also integral to recovery, aiming to improve function and alleviate symptoms [8].

Previous case reports have highlighted osteomyelitis of the mandible complicated by pathological fractures, demonstrating varying clinical presentations and diverse management approaches tailored to individual cases [1-11].

In this case, we report a patient presented with chronic mandibular symptoms. Histopathological analysis of an incisional biopsy revealed a benign fibro-osseous lesion with chronic inflammation and fibrosis, consistent with chronic osteomyelitis. Imaging via CT scan suggested chronic recurrent multifocal osteomyelitis (CRMO) as a differential diagnosis, alongside potential neoplastic processes. The treatment plan involved multiple surgeries and a mandibular segmental resection was planned based on clinical and radiological findings. However, intraoperative exploration revealed an unexpected outcome: the mandibular bone appeared structurally healthy, with normal bleeding patterns and compact bone, but lacking significant spongiosa. This finding led to a reassessment of the surgical strategy, emphasizing the importance of intraoperative evaluation in guiding treatment decisions.

## Case presentation

Patient history

An eight-year-old female patient reported to the Department of Oral and Maxillofacial Surgery with a complaint of progressive swelling of the lower jaw on the right side for the past six months. There was no history of trauma, paresthesia, or skin lesions, and the patient had no significant past medical or family history.

Her dental history included the extraction of a tooth followed by incision and drainage due to swelling. Despite these interventions, the swelling persisted, and the parents reported a progressive limitation in jaw opening. A computed tomography (CT) scan of the facial bones was performed at a different facility before the department visit. However, no biopsy had been conducted previously.

Clinical and radiographic examination

**Figure 1:**
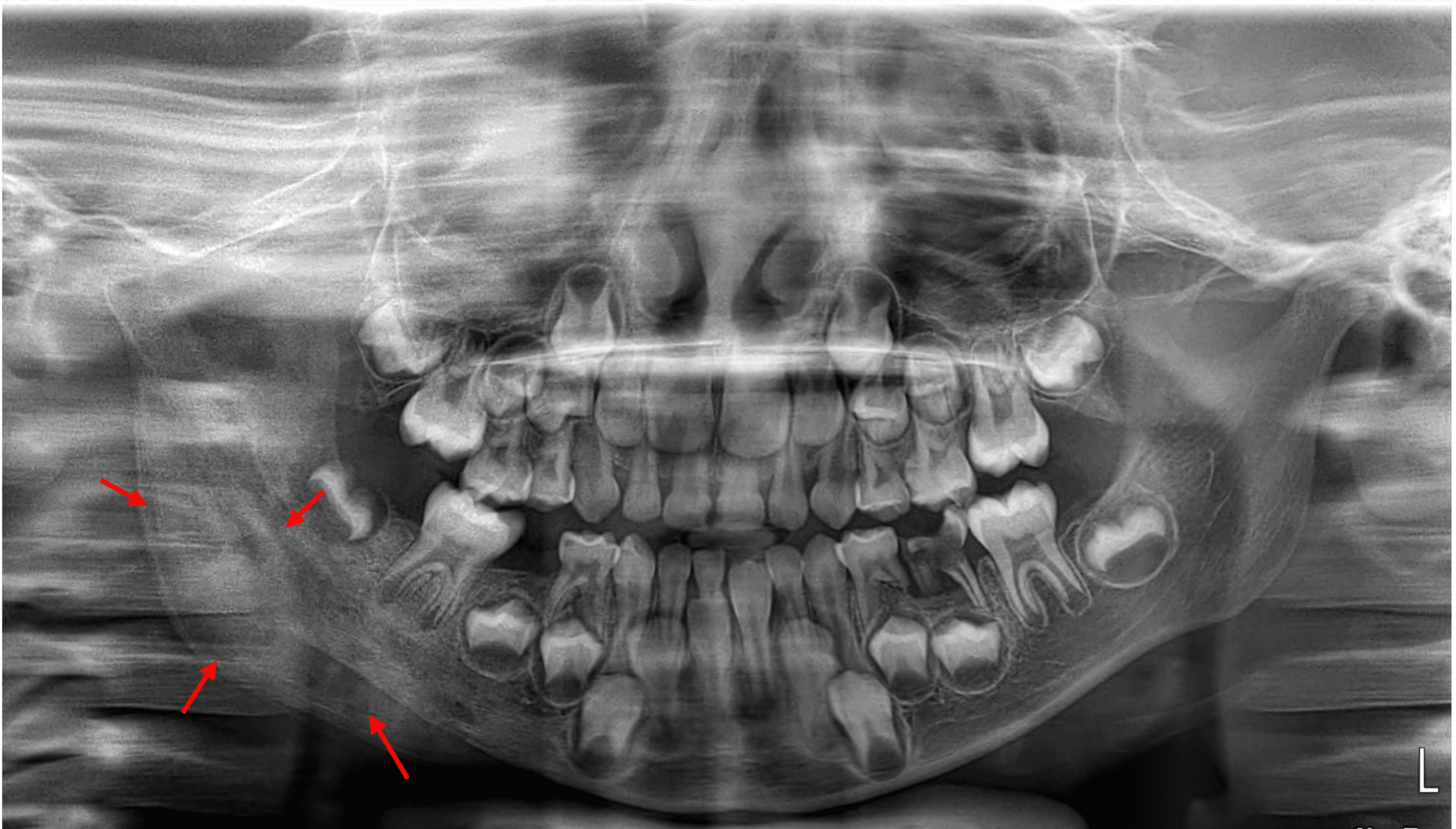
Orthopantomogram showing a radiopaque ossified lesion in the right mandible (red arrows).

Extraoral examination revealed a right facial swelling with a bony-hard consistency. There was no mobility of the teeth, and no palpable regional lymph nodes were noted. The patient presented with limited mouth opening, measuring only 13 mm interincisally. An orthopantomogram (Figure 1) revealed a radio-opaque ossified lesion in the right mandible, with a differential diagnosis suggesting a giant cell lesion or an osseous lesion.

**Figure 2:**
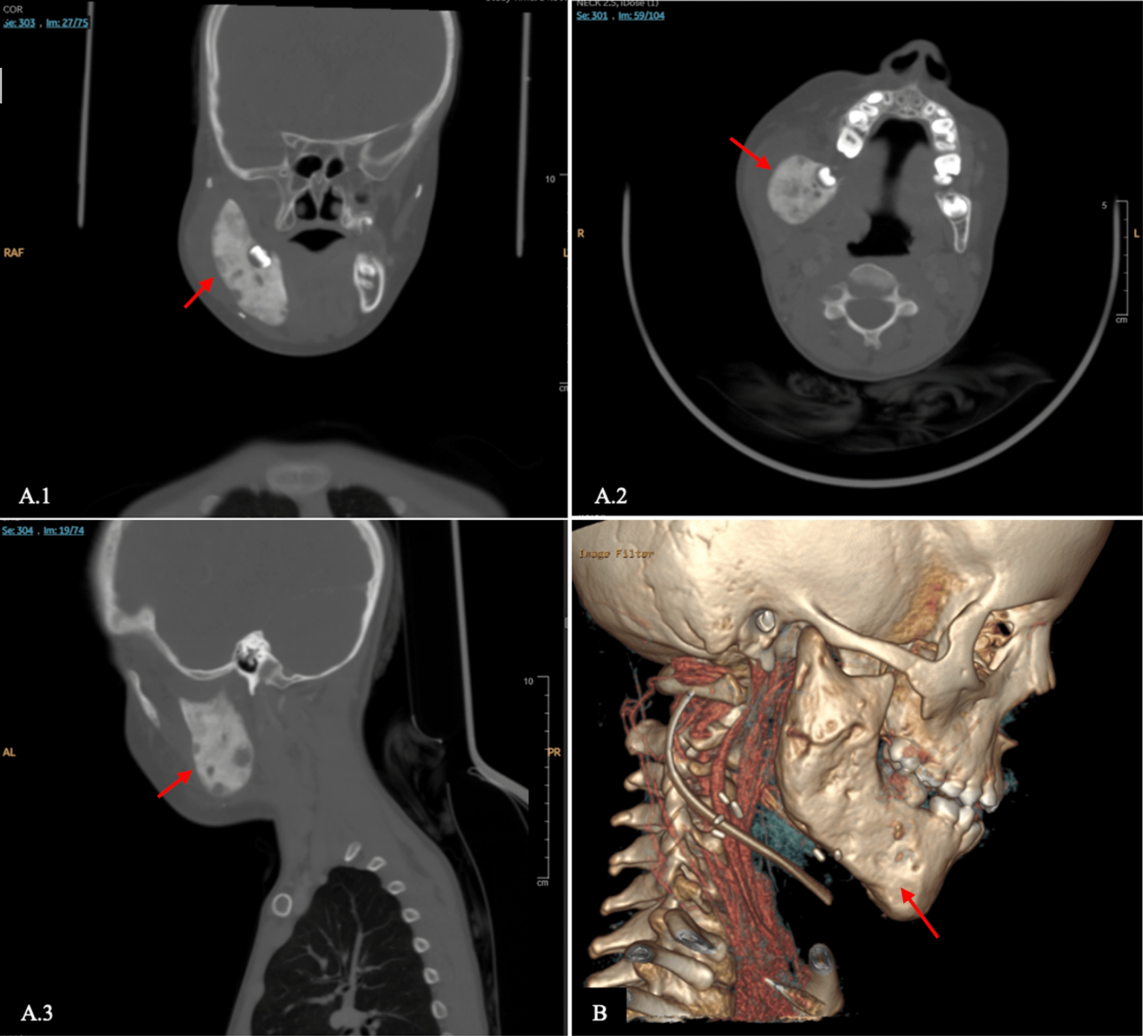
Computed tomography (CT) scan showing a lesion centered within the right mandibular ramus, extending to involve the coronoid and condylar processes, and anteriorly to the mandibular body near the symphysis menti. A.1: Pre-operative CT scan (coronal view); A.2: axial view; A.3: sagittal view. B: Three-dimensional CT. The red arrow shows the lesion.

A CT scan (Figure 2) showed a right infiltrative osseous lesion with extensive periosteal reaction and surrounding soft tissue mass. The lesion measures 32 × 28 × 48 mm in its maximum anteroposterior (AP), transverse (TR), and craniocaudal (CC) dimensions. It is centered within the right mandibular ramus, extending to involve the coronoid and condylar processes and anteriorly to the mandibular body near the symphysis menti. The lesion surrounds the roots of the teeth and contains internal cystic components. Focal areas of cortical breakthrough are observed. The associated soft tissue component involves the right masticator space and infratemporal fossa and appears inseparable from the surrounding muscles. This soft tissue component exerts a mass effect on the ipsilateral right parapharyngeal space, with mild edema. No significant cervical lymph node enlargement is noted. The oral cavity appears unremarkable, with no focal lesions identified. There is prominence of adenoid lymphoid tissue in the nasopharynx, causing moderate airway narrowing. The retropharyngeal and left parapharyngeal spaces are unremarkable. The thyroid gland and major salivary glands are within normal limits. The major neck vessels are patent. The visualized brain parenchyma and orbital structures appear within normal limits. The sinuses and mastoid air cells are well-aerated. Cuts through the upper lungs are clear. The right mandibular infiltrative osseous lesion shows an extensive smooth periosteal reaction and a surrounding soft tissue component. CRMO may be considered among the differential diagnoses, alongside potential neoplastic processes.

Surgical history

The patient underwent three surgical interventions for evaluation and management of the right mandibular lesion. The first procedure was an incisional biopsy, performed under general anesthesia. After securing the airway with an endotracheal tube, local anesthesia with 5 mL of lidocaine (1:100,000 epinephrine) was administered around the surgical site. The patient was prepped and draped in a sterile environment, and oral irrigation with chlorhexidine and saline was performed. A buccal incision was made in the retromolar area, followed by subperiosteal flap reflection. Bone samples were obtained for biopsy (Table 1) using a fissure bur, and deeper sections were collected with a bone curette. The wound was irrigated with normal saline, and hemostasis was achieved before closure with 4-0 Vicryl sutures (Ethicon, Inc., Bridgewater, USA).

**Table 1:** Histological features from the first surgery (incisional biopsy).

Specimen	Deception	Histological Findings
1	Right mandible lesion, excision	Mature trabecular bone with mild reactive changes and osteoblastic rimming

The second procedure involved exploration and biopsy from the right mandibular lesion. The patient was placed in the supine position, connected to monitors, and prepped and draped in a sterile manner. Local anesthesia (lidocaine with epinephrine 1:100,000) was administered. A curvilinear incision was made along a natural skin crease, extending from the right mastoid process across the midline. Subplatysmal skin flaps were elevated superiorly. The superficial layer of the deep cervical fascia was incised, and the anterior facial vein and facial artery were ligated and divided, exposing the facial node sent for biopsy (Table 2) and the periosteum sent for biopsy (Table 2) around the right mandible. The periosteum appeared thickened and fragile. It was incised, and subperiosteal dissection was performed from the right subcondylar region to the mental foramen anteriorly. The bone surface was shaved with osteotomes and a round bur was sent for biopsy (Table 2). At a deeper level, the soft tissue was sent for biopsy (Table 2), and inside the mandible, soft tissue was observed; it was curettage and sent for biopsy (Table 2). Its appearance was chondromatous. An intraoperative decision was made to stop the surgery and wait for the results of the incisional biopsy. Hemostasis was achieved, and copious irrigation with normal saline was performed. The neck was then closed in layers.

**Table 2:** Histological features from the second surgery (exploration and biopsy).

Specimen	Description	Histological Findings
1	Neck, right, right level 1B node, excision	One benign reactive lymph node, negative for malignancy
2	Oral cavity, periosteum right mandible, excision	Cauterized fibro-connective tissue
3	Oral cavity, mandibular bone (external layer), excision	Benign fibro-osseous lesion with fibrosis consistent with chronic fibrosing (late) osteomyelitis
4	Oral cavity, mandibular bone (core), excision	Benign fibro-osseous lesion with chronic inflammation and fibrosis consistent with chronic osteomyelitis
5	Oral cavity, mandibular bone (core - soft), excision	Benign fibro-osseous lesion with chronic inflammation and fibrosis consistent with chronic osteomyelitis

The third procedure was initially planned as a right mandibular segmental resection. However, it was modified intraoperatively based on the findings, and biopsies were taken (Table 3), and details are described in the treatment provided section.

**Table 3:** Histological features from the third surgery (bone shaving).

Specimen	Description	Histological Findings
1	Right neck lymph node, level 1B node, excision	Two benign lymph node
2	Right mandibular bone fragments excision	Benign fibro-osseous lesion with chronic inflammation and fibrosis consistent with chronic osteomyelitis

Histopathological examination and findings

The initial incisional biopsy (Table 1) shows fragments of mature bone trabeculae with mild reactive changes, osteoblastic rimming, and minimal inflammation. Features of fibrous dysplasia, osteosarcoma, giant cell-rich lesions, and other malignant neoplasms are not identified. Diagnostic considerations include an osteoid osteoma, which would require radiological correlation for additional characterization.

The second biopsies (Table 2) and the third (Table 3) collectively indicate a benign fibro-osseous lesion with features of chronic inflammation and fibrosis, consistent with chronic osteomyelitis. No evidence of malignancy or aggressive neoplastic processes is identified. Radiological and clinical correlation is recommended for a comprehensive diagnosis and management plan.

Management and treatment provided

**Figure 3:**
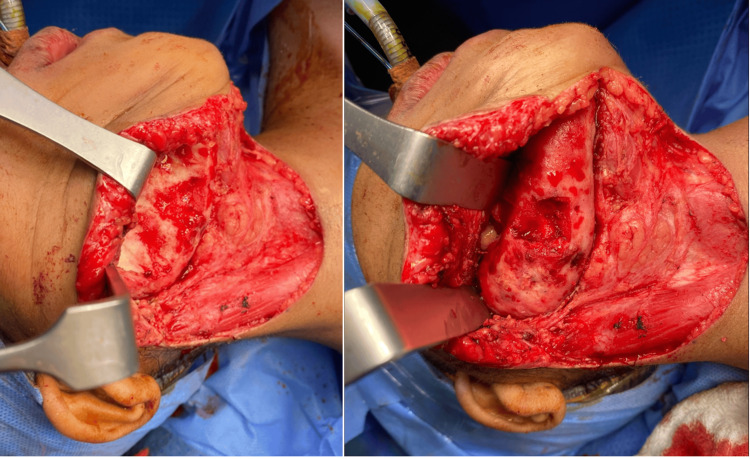
Intraoperative examination revealed the mandibular bone to be healthy, with normal bleeding observed.

Despite a trial of non-surgical management provided to the patient’s parents, which included treatment with clindamycin and prednisolone, the patient showed no significant improvement. A right mandibular segmental resection has been planned due to the limited mouth opening. Under general anesthesia, the patient could achieve a mouth opening of 4.5 cm. The patient was taken to the operating room in a supine position, monitored, and prepped in a sterile fashion. A curvilinear incision was made, following the previous scar from the right mastoid process to the midline, and subplatysmal flaps were elevated. The superficial layer of the deep cervical fascia was incised, allowing for dissection to the lower border of the mandible to protect the facial nerve. The periosteum of the mandible was then incised, and subperiosteal dissection was performed to expose the buccal surface of the mandible from the right premolar to the subcondylar area. Upon opening, the mandibular bone appeared healthy and bleeding, unlike the compacted appearance seen during the biopsy (Figure 3).

**Figure 4:**
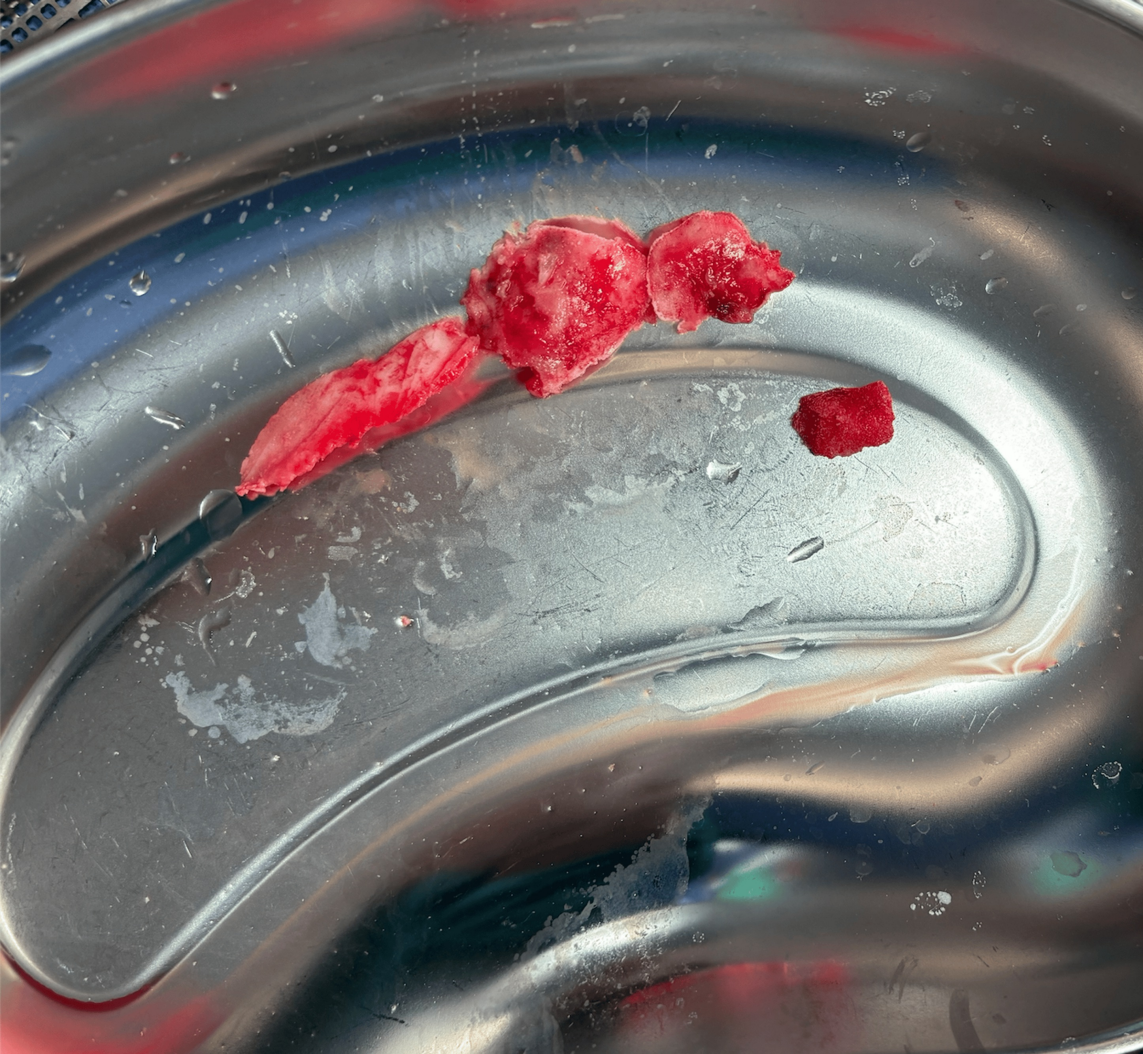
Specimen of the shaved excess mandibular bone along the lower border and buccal side, extending from the right premolar to the neck of the condyle.

A decision was made to shave the excess mandibular bone along the lower border and buccal side, from the right premolar to the neck of the condyle using a Stryker reciprocating saw (Stryker Corporation, Kalamazoo, Michigan, USA) and pineapple bur (Figure 4).

Intraorally, the temporalis muscle was released from the coronoid process, resulting in an improvement in the patient’s mouth opening, reaching 4.5 cm. After copious irrigation with normal saline, a Hemovac drain (Medtronic, Dublin, Ireland) was placed on the right neck and secured with silk sutures. The wound was closed in layers, including periosteum, platysma, subdermal layers, and skin, with subcuticular sutures. Preoperative antibiotics were administered, and venous thrombosis prophylaxis was deemed unnecessary.

Postoperative imaging, including a contrast-enhanced CT scan, revealed expected changes following the right mandible curettage and tooth extraction. A rim of fluid collection (measuring 0.7 x 0.5 x 5 cm) was identified at the surgical site, associated with soft tissue edema and fat stranding extending to the right hemiface. No new lytic lesions or drainable collections were seen. The scan also showed hypodensity in the right pterygoid and masseter muscles, as well as in the right gingiva, indicative of postoperative changes. Multiple enlarged cervical lymph nodes were noted, with the largest at level 2A, measuring 1.3 x 1.8 x 1.7 cm, likely reactive. Other findings included mild improvement in adenoid hypertrophy and normal findings in the left parotid gland, submandibular gland, thyroid, and bilateral mastoid air cells. There was no evidence of significant mucosal lesions or any major vascular abnormalities. The patient is recovering with no significant complications at this time, and the enlarged lymph nodes are expected to be reactive.

## Discussion

This case highlights the complexities of diagnosing and managing CSO, a rare inflammatory condition affecting the mandible [9]. Unlike acute osteomyelitis, CSO is characterized by non-suppurative chronic inflammation, fibrosis, and progressive sclerosis, with a limited understanding of its etiology. The patient’s clinical presentation of progressive jaw swelling, limited mouth opening, and persistent symptoms despite prior interventions aligns with the classic features of CSO [11,12].

Radiological findings, including periosteal reaction and bone sclerosis, were crucial in narrowing the differential diagnosis [5]. However, the final diagnosis required histopathological correlation to exclude malignancies such as osteosarcoma. This case underscores the importance of combining imaging modalities, histology, and clinical evaluation to establish a definitive diagnosis. The CT findings suggested CRMO as a differential diagnosis, a rare condition often presenting with multifocal bone lesions and periosteal reactions but lacking a bacterial origin. The choice of imaging modality should be carefully adapted to the clinical context, taking into account factors such as the region of interest, the level of detail needed, and whether soft tissue visualization is required. While cone-beam computed tomography (CBCT) is effective for high-resolution bone imaging, CT is more suitable for comprehensive assessment in cases of extensive osteomyelitis [3].

The decision to modify the surgical approach during the procedure highlights the role of intraoperative findings in guiding treatment. Although a segmental mandibular resection was initially planned, the observation of healthy, bleeding bone during exploration prompted a more conservative approach to bone shaving. This decision was pivotal in preserving mandibular integrity while addressing pathological bone overgrowth. Additionally, the patient had intact temporomandibular joint (TMJ) function and demonstrated good mouth opening, further supporting the success of the management. Postoperatively, imaging revealed expected changes, including fluid collection and soft tissue edema, consistent with surgical trauma. The absence of new lytic lesions or significant collections provided reassurance about the effectiveness of the intervention. The reactive nature of the enlarged cervical lymph nodes further supported the inflammatory rather than infectious or malignant origin of the disease.

This case shows the challenges of managing condensing sclerosing osteitis, particularly in refractory cases. Despite prior treatment with clindamycin and corticosteroids, the patient’s condition did not improve significantly, necessitating surgical intervention. The use of antibiotics and steroids in CSO management is based on the disease’s suspected low-grade bacterial infection and chronic inflammatory process [4,5].

Antibiotics are employed to target potential bacterial pathogens, such as slow-growing *Propionibacterium* species, which may contribute to persistent inflammation and bone sclerosis. Prolonged antibiotic therapy, often lasting three months or more, aims to reduce bacterial load, prevent disease progression, and provide symptomatic relief, although its effects are frequently transient [4].

Corticosteroids are used to manage the inflammatory component of CSO, offering rapid symptom relief by suppressing immune-mediated inflammation, thereby reducing pain and swelling during exacerbations. A typical course of corticosteroids involves an initial high dose, followed by gradual tapering. This approach helps extend the intervals between relapses and reduces their severity [4,5]. When combined, antibiotics and steroids address both infection and inflammation, providing a comprehensive approach to managing CSO. However, relapses remain common, and refractory cases may require surgical intervention. The observed improvement in jaw mobility following the release of the temporalis muscle underscores the functional benefits of timely surgical management.

In summary, this case contributes to the growing body of literature on CSO by illustrating the diagnostic and therapeutic challenges associated with this condition [9-12]. It underscores the importance of a multidisciplinary approach, incorporating imaging, histopathology, and surgical expertise to optimize outcomes. Further studies are needed to establish standardized treatment protocols for CSO, particularly in pediatric populations, to improve long-term prognosis and quality of life.

## Conclusions

CSO is a rare condition that poses significant diagnostic and therapeutic challenges due to its overlapping features with other pathological entities. This case of mandibular CSO underscores the importance of a comprehensive approach involving clinical assessment, advanced imaging, and histopathological analysis to exclude malignancy and establish an accurate diagnosis. The modification of the surgical plan based on intraoperative findings demonstrates the value of flexibility and precision in managing such cases.

Despite prior pharmacological treatments, the patient required surgical intervention to achieve symptomatic relief and functional improvement, particularly in jaw mobility. Postoperative findings suggest a favorable outcome with no evidence of new pathological lesions or complications. This case highlights the need for further research to refine diagnostic criteria and treatment protocols for CSO, especially in pediatric patients, to ensure timely interventions and better long-term outcomes.
